# Factors Associated with Attitudes towards Preventing Head and Neck Cancer through HPV Vaccination in Poland: A Nationwide Cross-Sectional Survey in 2021

**DOI:** 10.3390/vaccines10040632

**Published:** 2022-04-18

**Authors:** Wojciech Pinkas, Mateusz Jankowski, Waldemar Wierzba

**Affiliations:** 1 Central Clinical Hospital of the Ministry of the Interior and Administration in Warsaw, 02-507 Warsaw, Poland; 2 UHE Satellite Campus in Warsaw, University of Humanities and Economics in Łódź, 01-513 Warsaw, Poland; waldemar.wierzba@cskmswia.gov.pl; 3 School of Public Health, Centre of Postgraduate Medical Education, 01-826 Warsaw, Poland; mjankowski@cmkp.edu.pl

**Keywords:** human papillomavirus, HPV vaccine, HPV infection, head and neck cancers, vaccine hesitancy, vaccines, Poland

## Abstract

Human papillomavirus (HPV) infection is a risk factor for head and neck cancers (HNC). HPV-related head and neck cancers are preventable through vaccination. This study aimed to assess the attitudes towards HPV vaccination among adults in Poland, with particular emphasis on preventing HPV-related HNC, as well as identifying factors associated with a willingness to vaccinate children against HPV. This cross-sectional survey was carried out in November 2021 on a nationwide, representative sample of 1082 adults in Poland. The computer-assisted web interview (CAWI) technique was used. Only 42.5% of respondents were aware that HPV infection is a sexually transmitted disease. Less than one fourth of respondents (23.8%) indicated vaccination as an HPV infection prevention method and 51.9% of respondents correctly indicated HPV vaccine-eligible populations. Only 48.1% of respondents declared positive attitudes towards HPV vaccinations and declared that they would vaccinate their child against HPV. Males (OR: 1.43, 95% CI: 1.11–1.85; *p* < 0.01), respondents who did not have children (OR: 1.50, 95% CI: 1.04–2.14; *p* < 0.05), as well as those who had received a higher education (OR: 1.43, 95% CI: 1.11–1.85; *p* < 0.01), had greater odds of indicating positive attitudes towards HPV vaccinations. This study revealed a low level of public awareness of HPV vaccination as a cancer prevention method in Poland.

## 1. Introduction

Human papillomavirus (HPV) is a common viral infection [1,2]. The route of HPV transmission is primarily through sexual contact (vaginal, anal, or oral sex) [1,2,3,4]. HPV can also be transmitted by less intimate skin-to-skin contact (e.g., petting) [5]. It is estimated, that approximately 75% of sexually active men and women may acquire an HPV infection, at some point in their lives [6]. 

Most HPV infections are asymptomatic and clear spontaneously [1,2,7]. However, some types of HPV may lead to serious diseases [7]. There are more than 200 types of HPV, of which at least 14 types are oncogenic [2]. HPV has been found to cause cancer of the cervix, anus, penis, vulva, vagina, and oropharynx [8]. 

Human papillomavirus (HPV) causes more than 5% of cancers worldwide, including all cervical cancers and an increasing proportion of oropharyngeal cancers [9]. The HPV-16 and HPV-18 types account for approximately 70% of cervical cancers [10]. Moreover, the HPV-16 type accounts for approximately 90% of HPV-positive oropharyngeal cancers (OPCs) [10,11]. 

Epidemiological data showed that the incidence of HPV-related head and neck cancer (HNC) is increasing [12,13,14,15]. HPV-associated head and neck cancers have different risk factors, clinical characteristics, and tumor biology when compared to tobacco/alcohol-associated head and neck cancers [16]. Male gender, white race, age of 40–59 years, having multiple lifetime sex partners, and having orogenital and oro-anal sex, are the most common risk factors for HPV-associated head and neck cancers [17]. HPV-associated head and neck cancers are characterized by a younger age at onset, basaloid/warty histopathology, as well as superior survival outcomes when compared with their HPV-negative counterparts [18]. HPV-associated malignancies of the head and neck arise mainly in oropharyngeal sites, such as tonsils, the base of the tongue, and oropharynx; other oral sites, such as the ventrolateral tongue, gingivae, cheek, palate, and floor of the mouth are mainly attributed to tobacco use and alcohol consumption [15,19].

Vaccination is the most effective strategy for preventing HPV infection [20]. In 2006, the first HPV vaccine was introduced to the US and European markets [21]. Initially, the HPV vaccination programs aimed at genital, HPV-related lesions, and cervical cancer prevention. However, HPV vaccines have been demonstrated to be highly effective in preventing oral infections with HPV-16 and HPV-18—the types most found in the oropharynx [22]. Currently, available evidence suggests that existing HPV vaccines may well prevent HPV-associated oropharyngeal cancer [22,23,24,25,26].

HPV vaccination and HPV-based screening are cost-effective strategies for preventing cancer worldwide [27,28]. There are currently three HPV vaccines licensed in Europe, and all contain virus-like-particles (VLPs) of HPV types 16 and 18 [21,28]. In 2008, the European Centre for Disease Prevention and Control (ECDC) recommended to the European Union (EU) Member States to implement local HPV vaccination programs [21]. The implementation of HPV vaccination programs has varied considerably across the EU (mostly due to the will and funding availability in individual countries) [28]. In general, HPV vaccines are given in a two-dose regimen over a six-month period for individuals aged 9 to 15 years old and in three doses to individuals aged 16 years or older, with significant differences in vaccine schedules across the EU countries [28]. Findings from the analysis of HPV vaccination coverage, policies, and practical implementation in 21 European countries showed significant disparities in vaccination coverage rates [29]. In 10 countries (Belgium, Finland, Hungary, Iceland, Malta, Norway, Portugal, Spain, Sweden, the United Kingdom), the HPV vaccination coverage rate in girls aged 9–15 years exceeded 70% [29]. The lowest vaccination coverage rates (≤30%) were reported in Bulgaria, France, Greece, and Poland [29].

Poland is a country with one of the lowest HPV-vaccination coverage rates among the EU member states [29]. Since 2008, HPV vaccination has been recommended in the National Immunization Program for girls aged 11–12 years [21,28]. Moreover, in 2010, the Polish Pediatric Society also recommended HPV vaccines for girls aged 13–18 years who had not been previously vaccinated. As of January 2022, HPV vaccination is recommended in the Polish Preventive Vaccination Plan for both girls and boys before sexual initiation and according to the schedule recommended by manufacturers [30]. The coverage of Polish teenagers vaccinated against HPV is estimated to be between 7.5–10% [21,28]. 

In Poland, HPV vaccination is voluntary. There is an extra charge (cost of the vaccine—approximately 30 EUR) for prophylactic vaccination against HPV in primary healthcare settings (general practitioners or pediatricians) [21,28,30]. However, in some regions, HPV vaccines are available free of charge for teenage girls/children, but only in preventive programs run by local authorities [31]. There is no publicly funded HPV vaccination program for adults.

Data on public awareness of HPV vaccination and HPV infection prevention methods in Poland are very limited [32,33,34,35]. In recent years, there were educational campaigns on HPV and cervical cancer. However, up till now, public attitudes towards HPV vaccination as a prevention method against head and neck cancer have not been examined.

We aimed to assess the attitudes towards HPV vaccinations among adults in Poland, with a particular emphasis on preventing head and neck cancers through HPV vaccination, as well as to identify factors associated with a willingness to vaccinate children against HPV.

## 2. Materials and Methods

### 2.1. Study Desing and Population

This cross-sectional survey was carried out in November 2021 on a nationwide, representative sample of 1082 individuals aged 18 years and over in Poland. The computer-assisted web interview (CAWI) technique was used [36]. Data were collected by a specialized survey company (Nationwide Research Panel Ariadna [37]) on behalf of the research team, which provides the context of the survey. 

Respondents were selected from the Nationwide Research Panel Ariadna out of >100,000 registered and verified individuals aged 18 and over. The Nationwide Research Panel Ariadna is the biggest independent nationwide research panel in Poland, widely used in nationwide, representative cross-sectional surveys [38,39,40].

A non-probability quota sampling technique was used [37]. Participants were selected based on the stratification model, including gender, age, size of domicile, and the territorial distribution within administrative regions (16 voivodeships) in Poland. The stratification model included demographic data from Statistics Poland [41].

### 2.2. Measures

The research tool was a questionnaire developed for the purpose of this study. Questions on head and neck cancer, HPV infection, and attitudes towards the HPV vaccine were addressed. Questions also addressed personal characteristics, including gender, age, marital status, having children, educational level, size of place of residence, occupational status, and financial status. 

Self-reported knowledge about HPV infection. Respondents were asked about their awareness of HPV transmission, using the question: “How can you get infected with human papilloma virus (HPV)? Please select all that apply” with 6, mutually nonexclusive answers: by a kiss; by touching; by having sex; during natural childbirth; by having contact with the blood of an infected person; and difficult to tell/do not know. Moreover, respondents were asked about HPV infection prevention methods, using the question: “What do you think are the human papilloma virus (HPV) infection prevention methods? Please select all that apply” with 6, mutually nonexclusive answers: vaccination before the initiation of sexual contact; condom use; limiting the number of sexual partners and avoiding risky sexual behavior; other forms; infection with HPV cannot be prevented; and difficult to tell/do not know. Moreover, respondents were asked about the oncogenic potential of HPV, using the question: “What do you think are the risk factors for head and neck cancer?” with 10, mutually nonexclusive answers (including HPV infection as one of the answers [38]).

Self-reported knowledge about HPV vaccination. Respondents were asked about their knowledge of HPV vaccination recommendations (eligible groups), using the question: “In your opinion, who should be vaccinated against human papilloma virus (HPV)?” with four possible answers: girls before the initiation of sexual contact; boys before the initiation of sexual contact; boys and girls before the initiation of sexual contact; difficult to tell/do not know. Respondents who indicated “boys and girls before the initiation of sexual contact” were classified as those who were aware of the population eligible for the HPV vaccine.

Willingness to vaccinate children against HPV was defined using the question: “Would you like to vaccinate your child against human papilloma virus (HPV)?”. Respondents who indicated positive attitudes toward HPV vaccination by choosing the answers: “Definitely yes” or “Rather yes” were classified as those who declared a willingness to vaccinate their children against HPV.

### 2.3. Statistical Analysis

The data were analyzed with SPSS v. 28 (IBM, Armonk, NY, USA). The distribution of categorical variables was shown by frequencies and proportions. Statistical testing to compare categorical variables was completed using the independent samples chi-square (χ2) test.

Associations between sociodemographic factors such as gender, age, marital status, having children, place of residence, educational level, occupational status (active—currently employed or self-employed; passive—currently unemployed/pensioner/student), self-reported financial situation as well as self-reported knowledge about the oncogenic potential of HPV (head and neck cancer risk factor), and awareness of HPV infection prevention methods were analyzed using the logistic regression analyses. Vaccination before the initiation of sexual contact, condom use, and limiting the number of sexual partners and avoiding risky sexual behavior were considered separately as dependent variables in the model. These variables were selected because they are some of the most common HPV infection prevention methods that are well described in the literature.

Additionally, similar logistic regression analyses were carried out for the associations between sociodemographic factors and attitudes towards HPV vaccination (“positive attitudes towards HPV vaccination”), as well as knowledge about HPV vaccination recommendations (“awareness of population eligible for the HPV vaccine”). In univariate logistic regression analyses, all variables were considered separately. In multivariate logistic regression analyses, all sociodemographic variables were included. 

The strength of association was measured by the odds ratio (OR) and 95% confidence intervals (95% CI). The level of statistical significance was set at alpha 0.05. 

### 2.4. Ethics

This study was performed in line with the principles expressed in the Declaration of Helsinki. The study protocol was revised and approved by the Ethics Review Board at the Central Clinical Hospital of the Ministry of the Interior and Administration in Warsaw, Warsaw, Poland (approval number 131/2021). Informed consent was obtained from all the subjects involved in the study.

## 3. Results

### 3.1. Subjects’ Characteristics

Completed questionnaires were received from 1082 individuals (52.6% females), aged 18–86 years (mean age 44.5 ± 16.1). Detailed characteristics of the study population is presented in Table 1. 

### 3.2. Respondents’ Knowledge about HPV Infection and HPV Vaccination

Only one third (32.5%) of respondents were aware of the oncogenic potential of HPV and indicated HPV infection as a risk factor for head and neck cancer (Table 2). Most of the respondents indicated sex (42.5%) and contact with the blood of an infected person (42.4%) as the modes of transmission of HPV. The most identified HPV prevention methods were condom use (44.4%) and limiting the number of sexual partners and avoiding risky sexual behavior (44.4%). Less than one fourth of respondents indicated vaccination as an HPV infection prevention method. More than one third of respondents (35.1%) declared a lack of knowledge on HPV prevention methods (responses “difficult to tell” or “do not know”). Over half of the respondents (51.9%) correctly indicated that both boys and girls before the initiation of sexual contact should receive the HPV vaccine (eligible populations). Only 48.1% of respondents declared positive attitudes towards HPV vaccination and declared that they would vaccinate their child against HPV (20.5%—“definitely yes”; 27.6%—“rather yes”). Details are presented in Table 2.

### 3.3. Awareness of HPV Infection Prevention Methods by Sociodemographic Factors

Females compared to males more often indicated condom use (50.1% vs. 38.0%; *p* < 0.001) or limiting the number of sexual partners and avoiding risky sexual behavior (49.7% vs. 38.4%; *p* < 0.001) as a method of preventing HPV infection. The percentage of respondents who indicated vaccination before sexual initiation as a method of preventing HPV infection was significantly higher among those who lived in larger cities compared to those who lived in small cities (below 20,000 residents) or rural areas (Table 3). Respondents who had received a higher education were more aware of HPV infection prevention methods. There were no statistically significant differences in the respondents’ knowledge of the HPV infection prevention methods by age, marital status, having children, occupational status, and self-reported financial situation (Table 3).

### 3.4. Attitudes towards HPV Vaccination and Knowledge about HPV Vaccination Recommendations by Sociodemographic Factors

The percentage of respondents, who correctly indicated “boys and girls before the initiation of sexual contact” as eligible for the HPV vaccine, increased with age (Table 4). Moreover, respondents who had received a higher education, those with passive occupational status, as well as those who were aware of the oncogenic potential of HPV, indicated correct populations eligible for the HPV vaccine significantly more often. Females, respondents who had a higher education, respondents with good or very good self-reported financial situation, as well as those who were aware of the oncogenic potential of HPV, more often indicated positive attitudes towards HPV vaccination. Details are presented in Table 4.

### 3.5. Factors Associated with Awareness of HPV Infection Prevention Methods and HPV Vaccination

The results of the univariate and multivariate regression analyses are presented in Table 5 and Table 6. When adjusted for all covariables, females had higher odds of indicating that condom use (OR: 1.70, 95% CI: 1.31–2.19; *p* < 0.001) or limiting the number of sexual partners and avoiding risky sexual behavior (OR: 1.59, 95% CI: 1.23–2.06; *p* < 0.001) are the HPV infection prevention methods. Respondents aged 30–44 had higher odds of indicating that condom use is an HPV infection prevention method (OR: 1.56, 95% CI: 1.02–2.38; *p* < 0.05). Those respondents who lived in cities with more than 500,000 residents had higher odds of indicating that vaccination before the initiation of sexual contact (OR: 2.08, 95% CI: 1.32–3.28, *p* < 0.01) or condom use (OR: 1.51, 95% CI: 1.04–2.19; *p* < 0.05) are HPV infection prevention methods compared to those who lived in rural areas. Respondents who had a higher education had greater odds of being aware that vaccination before the initiation of sexual contact is an HPV infection prevention method (OR: 1.62, 95% CI: 1.21–2.22; *p* < 0.001). Respondents who were aware of the oncogenic potential of HPV (head and neck cancer risk factor) had higher odds of being aware of HPV infection prevention methods. Details are presented in Table 5.

Respondents who had received a higher education had higher odds of being aware of the population eligible for the HPV vaccine (OR: 1.34, 95% CI: 1.03–1.73; *p* < 0.05) as well as indicating positive attitudes towards HPV vaccination (OR: 1.43, 95% CI: 1.11–1.85; *p* < 0.01). Respondents who self-declared a rather good, good, or very good financial situation (OR: 0.69, 95% CI: 0.49–0.99; *p* < 0.05) or moderate financial situation (OR: 0.69, 95% CI: 0.48–0.98) had lower odds of being aware of the population eligible for the HPV vaccine (Table 6). Males (OR: 1.43, 95% CI: 1.11–1.85; *p* < 0.01) as well as those respondents who did not have children (OR: 1.50, 95% CI: 1.04–2.14; *p* < 0.05) had higher odds of indicating positive attitudes towards HPV vaccination. Respondents who lived in cities had higher odds of indicating positive attitudes towards HPV vaccination compared to those who lived in rural areas (Table 6). Respondents who were aware of the oncogenic potential of HPV (head and neck cancer risk factor) had higher odds of being aware of the population eligible for the HPV vaccine (OR: 2.56, 95% CI: 1.95–3.37; *p* < 0.001) as well as indicating positive attitudes towards HPV vaccination (OR: 1.95, 95% CI: 1.49–2.54; *p* < 0.001). Details are presented in Table 6.

## 4. Discussion

To the best of the authors’ knowledge, this is the most up-to-date study on the attitudes towards preventing head and neck cancers through HPV vaccination in a representative sample of adults in Poland. This study revealed a low level of public awareness regarding HPV vaccinations. Only 42.5% of respondents were aware that HPV infection is a sexually transmitted disease and 42.4% incorrectly indicated that HPV infection is a bloodborne disease. Less than one fourth of respondents correctly indicated that HPV can be prevented through vaccination, and approximately half of respondents correctly indicated HPV vaccine-eligible populations. Approximately half of the respondents declared positive attitudes towards HPV vaccinations. Public attitudes towards preventing head and neck cancer through HPV vaccination differed by sociodemographic factors, of which the educational level was the most important factor. 

According to the Polish National Cancer Registry, in 2015, 50% of newly diagnosed oropharyngeal cancers were attributed to HPV infection [42]. Despite the high burden of HPV-related cancers, in this study, only one third of respondents were aware of the oncogenic potential of HPV (head and neck cancer risk factor). There is varying knowledge even among health practitioners [32,33]. Jeruzal-Świątecka and Pietruszewska showed that more than 80% of medical students and approximately 56% of non-medical students were aware of the oncogenic potential of HPV infection, but less than 40% were aware that HPV may cause cancers other than cervical [32]. Jackowska et al. showed that 7% of ENTs, 20% of GPs, and 10% of trainees were not aware of HPV-related oropharyngeal diseases [33]. Awareness of the oncogenic potential in Poland is relatively low, which may lead to a low HPV vaccination coverage rate and may affect physicians’ willingness to inform patients about the possibility of HPV vaccinations.

In this study, less than half of the respondents (42.5%) correctly indicated sexual contact as a route of HPV transmission. We observed a relatively high percentage of respondents who incorrectly believed that HPV may be transmitted through blood (42.5%), by kiss (20.5%), or by touch (7.4%). A similar phenomenon was observed by Smolarczyk et al. in a group of parents in Poland [34]. In 2018, 78.9% of parents believed that HPV may be transmitted through blood contact, 90% indicated that HPV may be transmitted by a kiss, and 13.3% by touch [34]. Awareness of HPV transmission routes is crucial to implementing preventive measures. Educational campaigns on HPV transmission routes are needed.

In this study, limiting the number of sexual partners and avoiding risky sexual behavior as well as the use of condoms (44.4%) were the most recognized HPV infection prevention methods, wherein the percentage of respondents who were aware of these methods was significantly higher among females. In the study by Smolarczyk et al., 63.3% of parents in Warsaw, Poland indicated the use of condoms as an HPV infection prevention method and 70.6% indicated limiting the number of sexual partners as an HPV prevention method [34]. The differences between our study and the study by Smolarczyk et al. may result from different populations included in the study (a representative sample of adults in Poland vs. a convenience sample of parents in Warsaw). In our study, more than one third of respondents declared a lack of knowledge about HPV infection prevention methods. Most of the educational campaigns on HPV infection in Poland were focused on HPV-related cervical cancers and were led by gynecologists, so the target population was females. A further campaign should also include the role of HPV infection in oropharyngeal diseases and should address the general population.

In this study, only one quarter of respondents indicated vaccinations as an HPV prevention method. Respondents with a higher education, those who lived in cities with more than 500,000 residents, as well as those who were aware of the oncogenic potential of HPV (head and neck cancer risk factor), were more aware of the HPV vaccine as an infection prevention method. Our findings indicate that there is a need to increase the level of knowledge about HPV vaccinations among Poles with a lower level of education, as well as those who live in rural areas. Local governments, as well as local non-governmental organizations and associations (especially in rural areas), can be involved in education on HPV vaccinations. 

According to the Polish Preventive Vaccination Plan, HPV vaccination is recommended for girls and boys before sexual initiation [30]. In this study, only 51.9% of respondents correctly indicated HPV vaccine-eligible populations. There was no significant impact of gender, age, or place of residence on public awareness of HPV vaccine-eligible populations. A lack of significant differences by socioeconomic factors (except the educational level) suggests that other factors may have an impact on public awareness of HPV vaccine-eligible populations. Physicians play an essential role in promoting vaccination. Most of the medical doctors in Poland support HPV vaccination [35]. However, in 2018, only 65% of gynecologists and 51.2% of pediatrists informed patients about the possibility of vaccinating their daughters against HPV [35]. We can hypothesize that physicians and their attitudes towards HPV vaccination, especially their willingness to inform patients about the possibility of vaccination, play a crucial role in promoting HPV vaccinations.

Regular monitoring of public awareness towards the HPV vaccine is an essential part of vaccination policy. Identification of public concerns and hesitant attitudes allows for the promotion of educational campaigns that build vaccine confidence. Gańczak et al. showed that between 2013–2014, 85.1% of parents of high school students from three selected high schools in Zgorzelec, Poland, declared a willingness to vaccinate their children against HPV [43]. In 2018, Smolarczyk et al. showed that 55% of parents from Warsaw, Poland, would have vaccinated their children (if the vaccination was covered by the government) [34]. In this study, less than half of the respondents (48.1%) declared that they would vaccinate their child against HPV. The differences between our study and study by Gańczak et al. [43] or Smolarczyk et al. [34] may result from different study populations, selection (representative sample vs. local, convenience sample), as well as the study year. Nevertheless, in each of the studies, the proportion of respondents who declared a willingness to vaccinate their children against HPV is multiple times higher than the real HPV vaccination coverage rate in Poland and indicates potential barriers to accessing HPV vaccinations in Poland. Logistic barriers such as the availability of vaccines, access to vaccination points, financial barriers (lack of reimbursement), as well as a lack of knowledge about HPV infection and vaccines, are considered major factors limiting the widespread implementation of HPV vaccination programs in Poland [34]. 

This study has practical implications for vaccination policies. The proportion of respondents who declared their willingness to vaccinate their children against HPV was five times lower than the HPV vaccination coverage rate among teenagers in Poland, which may indicate systemic barriers to receiving HPV vaccinations. Further activities are needed to ensure easy access to HPV vaccines for those individuals who intend to vaccinate their children. Organizational barriers, as well as financial barriers, are considered the most important factors [29,34]. Public health activities (e.g., full public funding of HPV vaccines and pharmacy-located HPV vaccinations) are needed to ensure easy access to HPV vaccines for those individuals who intend to vaccinate their children against HPV. Moreover, otolaryngologists should be actively involved in cancer prevention programs. The head and neck cancer burden in Poland (including HPV-related cancers) is increasing, so otolaryngologists should also educate their patients on HPV prevention methods.

Our findings suggest that there is an urgent need to conduct a nationwide information campaign on HPV infection prevention methods and the health risk of HPV-related diseases. In Poland, most educational campaigns on HPV infections were led by obstetricians or gynecologists and were addressed to females. Further educational campaigns should target all genders. 

This study had several potential limitations. This study was carried out using the CAWI research method, which excludes the possibility of interaction with the respondents. Therefore, the ability to assess the competencies of the respondents, for example, whether they sufficiently understand the questions asked, is impossible. Second, we cannot exclude non-response bias. However, similar methods were used in previously published papers [38,39,40]. Moreover, questions on barriers restricting access to HPV vaccinations, as well as the potential impact of religious beliefs on attitudes towards HPV vaccinations, were not included in this study. 

## 5. Conclusions

This study demonstrated the low public awareness of HPV infection prevention methods among adults in Poland. Less than one quarter of adults in Poland were aware that HPV infection can be prevented through vaccination, and only half of the Poles declared positive attitudes towards HPV vaccinations. Male gender, living in cities, and having a higher education were the most important factors associated with positive attitudes towards HPV vaccinations. The current study indicated significant gaps in the recognition of HPV vaccine-eligible populations. The presented data underscore the importance of adopting an educational campaign on HPV infection prevention methods, with particular emphasis on vaccinations. Otolaryngologists should be actively involved in the educational campaign on HPV vaccines, due to the significant burden of HPV-related head and neck cancers in Poland.

## Figures and Tables

**Table 1:** Characteristics of the study population.

Variable		Overall
		*n* = 1082 (100%)
Gender		
Female	569	52.6
Male	513	47.4
Age (years)		
18–29	217	20.1
30–44	311	28.7
44–59	289	26.7
60+	265	24.5
Marital status		
single	236	21.8
married	546	50.5
informal relationship	161	14.9
divorced	65	6.0
widowed	74	6.8
Having children		
Yes	697	64.4
No	385	35.6
Place of residence		
rural	343	31.7
city below 20,000 residents	130	12.0
city from 20,000 to 99,999 residents	238	22.0
city from 100,000 to 499,999 residents	207	19.1
city above 500,000 residents	164	15.2
Having higher education		
Yes	461	42.6
No	621	57.4
Occupational status		
active	666	61.6
passive	416	38.4
Self-reported financial situation		
good, or very good	165	15.3
rather good	307	28.4
moderate/difficult to tell	411	38.0
rather bad	97	9.0
bad or very bad	102	9.5

**Table 2:** Respondents’ knowledge about HPV infection and HPV vaccination, Poland, November 2021 (*n* = 1082).

Variable	*n*	% (95%CI)
Awareness of the oncogenic potential of HPV (head and neck cancer risk factor)		
Yes	352	32.5 (29.8–35.4)
No	730	67.5 (64.6–70.2)
How can you get infected with human papilloma virus (HPV)? *Please select all that apply*		
by a kiss	222	20.5 (18.2–23.0)
by touching	80	7.4 (6.0–9.1)
by having sex	460	42.5 (39.6–45.5)
during natural childbirth	75	6.9 (5.6–8.6)
by having contact with the blood of an infected person	459	42.4 (39.5–45.4)
difficult to tell/do not know	380	35.1 (32.3–38.0)
What do you think are the human papilloma virus (HPV) infection prevention methods? *Please select all that apply*		
vaccination before the initiation of sexual contact	258	23.8 (21.4–26.5)
condom use	480	44.4 (41.4–47.3)
limiting the number of sexual partners and avoiding risky sexual behavior	480	44.4 (41.4–47.3)
other form	5	0.5 (0.0–0.1)
infection with HPV cannot be prevented	31	2.9 (2.0–4.0)
difficult to tell/do not know	418	38.6 (35.6–41.6)
In your opinion, who should be vaccinated against human papilloma virus (HPV)?		
girls before the initiation of sexual contact	72	6.7 (5.3–8.3)
boys before the initiation of sexual contact	16	1.5 (0.9–2.4)
boys and girls before the initiation of sexual contact	562	51.9 (49.0–54.9)
difficult to tell/do not know	441	40.8 (37.9–43.7)
Would you like to vaccinate your child against human papilloma virus (HPV)?		
definitely yes	222	20.5 (18.2–23.0)
rather yes	299	27.6 (25.1–30.4)
definitely no	94	8.7 (7.2–10.5)
rather no	71	6.6 (5.2–8.2)
difficult to tell/do not know	396	36.6 (33.8–39.5)

**Table 3:** Awareness of HPV infection prevention methods by sociodemographic factors, Poland, November 2021 (*n* = 1082).

	Awareness of HPV Infection Prevention Methods—Percentage of Respondents Who Answered “Yes” by Sociodemographic Factors					
Variable	Vaccination before the Initiation of Sexual Contact		Condom Use		Limiting the Number of Sexual Partners and Avoiding Risky Sexual Behavior	
	*n* (*%*)	*p*	*n* (*%*)	*p*	*n* (*%*)	*p*
Gender						
Female	140 (24.6)	0.5	285 (50.1)	<0.001	283 (49.7)	<0.001
Male	118 (23.0)		195 (38.0)		197 (38.4)	
Age (years)						
18–29	48 (22.1)	0.5	99 (45.6)	0.2	105 (48.4)	0.6
30–44	81 (26.0)		152 (48.9)		132 (42.4)	
44–59	72 (24.9)		122 (42.2)		127 (43.9)	
60+	57 (21.5)		107 (40.4)		116 (43.8)	
Marital status						
single	65 (27.5)	0.3	107 (45.3)	0.6	110 (46.6)	0.9
married	120 (22.0)		248 (45.4)		237 (43.4)	
informal relationship	42 (26.1)		72 (44.7)		72 (44.7)	
divorced	17 (26.2)		24 (36.9)		29 (44.6)	
widowed	14 (18.9)		29 (39.2)		32 (43.2)	
Having children						
Yes	158 (22.7)	0.2	304 (43.6)	0.5	298 (42.8)	0.2
No	100 (26.0)		176 (45.7)		182 (47.3)	
Place of residence						
rural	71 (20.7)	0.002	143 (41.7)	0.1	136 (39.7)	0.1
city below 20,000 residents	22 (16.9)		62 (47.7)		55 (42.3)	
city from 20,000 to 99,999 residents	59 (24.8)		95 (39.9)		104 (43.7)	
city from 100,000 to 499,999 residents	48 (23.2)		95 (45.9)		103 (49.8)	
city above 500,000 residents	58 (35.4)		85 (51.8)		82 (50.0)	
Having higher education						
Yes	140 (30.4)	<0.001	227 (49.2)	0.005	224 (48.6)	0.02
No	118 (19.0)		253 (40.7)		256 (41.2)	
Occupational status						
active	172 (25.8)	0.05	307 (46.1)	0.1	300 (45.0)	0.6
passive	86 (20.7)		173 (41.6)		180 (43.3)	
Self-reported financial situation						
good, or very good	41 (24.8)	0.7	78 (47.3)	0.5	78 (47.3)	0.3
rather good	80 (26.1)		144 (46.9)		144 (46.9)	
moderate/difficult to tell	95 (23.1)		172 (41.8)		172 (41.8)	
rather bad	19 (19.6)		39 (40.2)		36 (37.1)	
bad or very bad	23 (22.5)		47 (46.1)		50 (49.0)	
Awareness of oncogenic potential of HPV (head and neck cancer risk factor)						
Yes	141 (40.1)	<0.001	214 (60.8)	<0.001	228 (64.8)	<0.001
No	117 (16.0)		266 (36.4)		252 (34.5)	

**Table 4:** Attitudes towards HPV vaccination and knowledge about HPV vaccination recommendations by sociodemographic factors, Poland, November 2021 (*n* = 1082).

Variable	Respondents Who Declared “Boys and Girls before the Initiation of Sexual Contact” as Eligible for the HPV Vaccine		Respondents Who Declared Positive Attitudes towards HPV Vaccination	
	*n* (%)	*p*	*n* (%)	*p*
Gender				
Female	302 (53.1)	0.4	254 (44.6)	0.02
Male	260 (50.7)		267 (52.0)	
Age (years)				
18–29	115 (53.0)	0.002	101 (46.5)	0.3
30–44	146 (46.9)		139 (44.7)	
44–59	138 (47.8)		141 (48.8)	
60+	163 (61.5)		140 (52.8)	
Marital status				
single	126 (53.4)	0.4	116 (49.2)	0.9
married	289 (52.9)		260 (47.6)	
informal relationship	82 (50.9)		77 (47.8)	
divorced	35 (53.8)		34 (52.3)	
widowed	30 (40.5)		34 (45.9)	
Having children				
Yes	361 (51.8)	0.9	325 (46.6)	0.2
No	201 (52.2)		196 (50.9)	
Place of residence				
rural	168 (49.0)	0.2	138 (40.2)	0.002
city below 20,000 residents	65 (50.0)		67 (51.5)	
city from 20,000 to 99,999 residents	119 (50.0)		111 (46.6)	
city from 100,000 to 499,999 residents	112 (54.1)		112 (54.1)	
city above 500,000 residents	98 (59.8)		93 (56.7)	
Having higher education				
Yes	259 (56.2)	0.02	251 (54.4)	<0.001
No	303 (48.8)		270 (43.5)	
Occupational status				
active	325 (48.8)	0.01	321 (48.2)	0.9
passive	237 (57.0)		200 (48.1)	
Self-reported financial situation				
good, or very good	82 (49.7)	0.08	90 (54.5)	0.01
rather good	160 (52.1)		162 (52.8)	
moderate/difficult to tell	202 (49.1)		170 (41.4)	
rather bad	63 (64.9)		52 (53.6)	
bad or very bad	55 (53.9)		47 (46.1)	
Awareness of oncogenic potential of HPV (head and neck cancer risk factor)				
Yes	236 (67.0)	<0.001	209 (59.4)	<0.001
No	326 (44.7)		312 (42.7)	

**Table 5:** Factors associated with awareness of HPV infection prevention methods (*n* = 1082).

	Factors Associated with Awareness of HPV Infection Prevention Methods					
Variable	Vaccination before the Initiation of Sexual Contact		Condom Use		Limiting the Number of Sexual Partners and Avoiding Risky Sexual Behavior	
	Univariate Logistic Regression	Multivariate Logistic Regression	Univariate Logistic Regression	Multivariate Logistic Regression	Univariate Logistic Regression	Multivariate Logistic Regression
	OR (95%CI)	aOR (95%CI)	OR (95%CI)	aOR (95%CI)	OR (95%CI)	aOR (95%CI)
Gender						
Female	1.09 (0.83–1.45)	1.08 (0.80–1.46)	1.64 (1.28–2.09) ***	1.70 (1.31–2.19) ***	1.59 (1.25–2.02) ***	1.59 (1.23–2.06) ***
Male	Reference	Reference	Reference	Reference	Reference	Reference
Age (years)						
18–29	1.04 (0.67–1.60)	0.86 (0.49–1.52)	1.24 (0.86–1.78)	1.25 (0.78–1.99)	1.20 (0.84–1.73)	1.10 (0.69–1.77)
30–44	1.29 (0.87–1.89)	1.21 (0.73–2.02)	1.41 (1.01–1.97) *	1.56 (1.02–2.38) *	0.95 (0.68–1.32)	0.98 (0.63–1.50)
44–59	1.21 (0.82–1.80)	1.24 (0.76–2.01)	1.08 (0.77–1.51)	1.12 (0.75–1.70)	1.01 (0.72–1.41)	1.01 (0.67–1.53)
60+	Reference	Reference	Reference	Reference	Reference	Reference
Marital status						
ever married	Reference	Reference	Reference	Reference	Reference	Reference
never married	1.31 (0.98–1.74)	1.42 (0.93–2.14)	1.05 (0.82–1.34)	0.94 (0.66–1.35)	1.10 (0.86–1.41)	0.93 (0.65–1.34)
Having children						
Yes	0.84 (0.63–1.12)	0.90 (0.59–1.37)	0.92 (0.72–1.18)	0.85 (0.59–1.22)	0.83 (0.65–1.07)	0.71 (0.49–1.02)
No	Reference	Reference	Reference	Reference	Reference	Reference
Place of residence						
rural	Reference	Reference	Reference	Reference	Reference	Reference
city below 20,000 residents	0.78 (0.46–1.32)	0.81 (0.47–1.40)	1.28 (0.85–1.91)	1.37 (0.90–2.10)	1.12 (0.74–1.68)	1.19 (0.77–1.83)
city from 20,000 to 99,999 residents	1.26 (0.85–1.87)	1.39 (0.91–2.11)	0.93 (0.66–1.30)	1.00 (0.70–1.43)	1.18 (0.85–1.65)	1.29 (0.90–1.84)
city from 100,000 to 499,999 residents	1.16 (0.76–1.75)	1.02 (0.65–1.58)	1.19 (0.84–1.68)	1.11 (0.77–1.61)	1.51 (1.06–2.14) *	1.38 (0.95–2.01)
city above 500,000 residents	2.10 (1.39–3.17) ***	2.08 (1.32–3.28) **	1.51 (1.04–2.19) *	1.51 (1.01–2.26) *	1.52 (1.05–2.21) *	1.44 (0.96–2.17)
Having higher education						
Yes	1.86 (1.40–2.47) ***	1.64 (1.21–2.22) ***	1.41 (1.12–1.80) **	1.26 (0.97–1.63)	1.35 (1.06–1.72) *	1.22 (0.94–1.59)
No	Reference	Reference	Reference	Reference	Reference	Reference
Occupational status						
active	1.34 (0.99–1.79)	1.06 (0.73–1.55)	1.20 (0.94–1.54)	1.00 (0.73–1.37)	1.08 (0.84–1.38)	1.01 (0.74–1.39)
passive	Reference	Reference	Reference	Reference	Reference	Reference
Self-reported financial situation						
rather good, good or very good	1.30 (0.87–1.92)	1.23 (0.80–1.89)	1.17 (0.84–1.63)	1.10 (0.77–1.57)	1.17 (0.84–1.63)	1.10 (0.77–1.57)
moderate/difficult to tell	1.12 (0.75–1.69)	1.16 (0.75–1.81)	0.95 (0.67–1.33)	0.95 (0.66–1.37)	0.95 (0.67–1.33)	0.94 (0.65–1.35)
rather bad, bad or very bad	Reference	Reference	Reference	Reference	Reference	Reference
Awareness of oncogenic potential of HPV (head and neck cancer risk factor)						
Yes	3.50 (2.62–4.68) ***	3.58 (2.65–4.84) ***	2.71 (2.08–3.51) ***	2.67 (2.04–3.49) ***	3.49 (2.67–4.55) ***	3.47 (2.64–4.55) ***
No	Reference	Reference	Reference	Reference	Reference	Reference

* *p* < 0.05; ** *p* < 0.01; *** *p* < 0.001.

**Table 6:** Factors associated with attitudes towards HPV vaccination and knowledge about HPV vaccination recommendations (*n* = 1082).

	Factors Associated with Attitudes towards HPV Vaccination and Knowledge about HPV Vaccination Recommendations			
Variable	Awareness of Population Eligible for the HPV Vaccine		Positive Attitudes towards HPV Vaccination	
	Univariate Logistic Regression	Multivariate Logistic Regression	Univariate Logistic Regression	Multivariate Logistic Regression
	OR (95%CI)	aOR (95%CI)	OR (95%CI)	aOR (95%CI)
Gender				
Female	1.10 (0.87–1.40)	1.05 (0.81–1.35)	Reference	Reference
Male	Reference	Reference	1.35 (1.06–1.71) *	1.43 (1.11–1.85) **
Age (years)				
18–29	Reference	Reference	Reference	Reference
30–44	0.79 (0.56–1.11)	0.83 (0.56–1.23)	0.93 (0.66–1.32)	0.92 (0.62–1.36)
44–59	0.81 (0.57–1.15)	0.88 (0.58–1.33)	1.09 (0.77–1.56)	1.25 (0.82–1.90)
60+	1.42 (0.99–2.04)	1.44 (0.90–2.28)	1.29 (0.90–1.84)	1.40 (0.88–2.23)
Marital status				
ever married	Reference	Reference	Reference	Reference
never married	1.03 (0.80–1.32)	1.12 (0.78–1.60)	1.03 (0.80–1.32)	0.88 (0.61–1.25)
Having children				
Yes	Reference	Reference	Reference	Reference
No	1.02 (0.79–1.30)	1.09 (0.76–1.56)	1.19 (0.93–1.52)	1.50 (1.04–2.14) *
Place of residence				
rural	Reference	Reference	Reference	Reference
city below 20,000 residents	1.04 (0.70–1.56)	1.03 (0.68–1.57)	1.58 (1.05–2.37) *	1.58 (1.04–2.40) *
city from 20,000 to 99,999 residents	1.04 (0.75–1.45)	1.03 (0.73–1.46)	1.30 (0.93–1.81)	1.34 (0.94–1.90)
city from 100,000 to 499,999 residents	1.23 (0.87–1.74)	1.05 (0.73–1.51)	1.75 (1.24–2.48) **	1.65 (1.14–2.38) *
city above 500,000 residents	1.55 (1.06–2.26) *	1.28 (0.85–1.91)	1.95 (1.34–2.84) ***	1.75 (1.17–2.61) **
Having higher education				
Yes	1.35 (1.06–1.71) *	1.34 (1.03–1.73) *	1.55 (1.22–1.98) ***	1.43 (1.11–1.85) **
No	Reference	Reference	Reference	Reference
Occupational status				
active	Reference	Reference	1.00 (0.78–1.27)	1.05 (0.77–1.43)
passive	1.39 (1.09–1.78) **	1.19 (0.87–1.62)	Reference	Reference
Self-reported financial situation				
rather good, good or very good	0.72 (0.52–1.01)	0.69 (0.49–0.99) *	1.16 (0.83–1.61)	1.12 (0.79–1.58)
moderate/difficult to tell	0.66 (0.47–0.94) *	0.69 (0.48–0.98) *	0.71 (0.51–1.00)	0.70 (0.49–1.00)
rather bad, bad or very bad	Reference	Reference	Reference	Reference
Awareness of oncogenic potential of HPV (head and neck cancer risk factor)				
Yes	2.52 (1.93–3.29) ***	2.56 (1.95–3.37) ***	1.96 (1.51–2.54) ***	1.95 (1.49–2.54) ***
No	Reference	Reference	Reference	Reference

* *p* < 0.05; ** *p* < 0.01; *** *p* < 0.001.

## Data Availability

Data are available upon reasonable request. The dataset used to conduct the analyses is available from the corresponding author upon reasonable request.

## References

[1] Brianti P., De Flammineis E., Mercuri S.R. (2017). Review of HPV-related diseases and cancers. New Microbiol..

[2] Cheng L., Wang Y., Du J. (2020). Human Papillomavirus Vaccines: An Updated Review. Vaccines.

[3] Centers for Disease Control and Prevention HPV and Oropharyngeal Cancer. https://www.cdc.gov/cancer/hpv/basic_info/hpv_oropharyngeal.htm.

[4] Nassif S.J., Steinwald P., Tracy J.C. (2022). Human Papillomavirus Vaccine and Head and Neck Cancer. JAMA Otolaryngol. Head Neck Surg..

[5] Petca A., Borislavschi A., Zvanca M.E., Petca R.C., Sandru F., Dumitrascu M.C. (2020). Non-sexual HPV transmission and role of vaccination for a better future (Review). Exp. Ther. Med..

[6] Bharti A.H., Chotaliya K., Marfatia Y.S. (2013). An update on oral human papillomavirus infection. Indian J. Sex Transm. Dis. AIDS.

[7] Crosbie E.J., Einstein M.H., Franceschi S., Kitchener H.C. (2013). Human papillomavirus and cervical cancer. Lancet.

[8] Saraiya M., Unger E.R., Thompson T.D., Lynch C.F., Hernandez B.Y., Lyu C.W., Steinau M., Watson M., Wilkinson E.J., Hopenhayn C. (2015). US assessment of HPV types in cancers: Implications for current and 9-valent HPV vaccines. J. Natl. Cancer Inst..

[9] Berman T.A., Schiller J.T. (2017). Human papillomavirus in cervical cancer and oropharyngeal cancer: One cause, two diseases. Cancer.

[10] International Agency for Research on Cancer (IARC), World Health Organization (WHO) (2012). GLOBOCAN 2012: Cervical Cancer Estimated Incidence, Mortality and Prevalence Worldwide in 2012.

[11] Dayyani F., Etzel C.J., Liu M., Ho C.H., Lippman S.M., Tsao A.S. (2010). Meta-analysis of the impact of human papillomavirus (HPV) on cancer risk and overall survival in head and neck squamous cell carcinomas (HNSCC). Head Neck Oncol..

[12] Chaturvedi A.K., Engels E.A., Pfeiffer R.M., Hernandez B.Y., Xiao W., Kim E., Jiang B., Goodman M.T., Sibug-Saber M., Cozen W. (2011). Human papillomavirus and rising oropharyngeal cancer incidence in the United States. J. Clin. Oncol..

[13] Licitra L., Zigon G., Gatta G., Sánchez M.J., Berrino F., EUROCARE Working Group (2008). Human papillomavirus in HNSCC: A European epidemiologic perspective. Hematol. Oncol. Clin. N. Am..

[14] Menezes F.D.S., Fernandes G.A., Antunes J.L.F., Villa L.L., Toporcov T.N. (2021). Global incidence trends in head and neck cancer for HPV-related and -unrelated subsites: A systematic review of population-based studies. Oral Oncol..

[15] Aldalwg M.A.H., Brestovac B. (2017). Human Papillomavirus Associated Cancers of the Head and Neck: An Australian Perspective. Head Neck Pathol..

[16] Benson E., Li R., Eisele D., Fakhry C. (2014). The clinical impact of HPV tumor status upon head and neck squamous cell carcinomas. Oral Oncol..

[17] Yakin M., Seo B., Hussaini H., Rich A., Hunter K. (2019). Human papillomavirus and oral and oropharyngeal carcinoma: The essentials. Aust. Dent. J..

[18] Gillison M.L., Chaturvedi A.K., Lowy D.R. (2008). HPV prophylactic vaccines and the potential prevention of noncervical cancers in both men and women. Cancer.

[19] Ragin C.C., Liu J.C. (2014). Epidemiology of HPV in head and neck cancer: Variant strains, discrete protein function. Molecular Determinants of Head and Neck Cancer.

[20] Savoy M. (2019). Vaccination is the Most Effective Strategy for HPV Prevention. Dela. J. Public Health.

[21] European Centre for Disease Prevention and Control (2008). Guidance for the Introduction of HPV Vaccines in EU Countries. https://www.ecdc.europa.eu/sites/default/files/documents/Guidance-on-HPV-vaccination-in-EU-countries2020-03-30.pdf.

[22] Herrero R., Quint W., Hildesheim A., Gonzalez P., Struijk L., Katki H.A., Porras C., Schiffman M., Rodriguez A.C., Solomon D. (2013). Reduced prevalence of oral human papillomavirus (HPV) 4 years after bivalent HPV vaccination in a randomized clinical trial in Costa Rica. PLoS ONE.

[23] Wierzbicka M., Józefiak A., Jackowska J., Szydłowski J., Goździcka-Józefiak A. (2014). HPV vaccination in head and neck HPV-related pathologies. Otolaryngol. Pol..

[24] Osazuwa-Peters N. (2013). Human papillomavirus (HPV), HPV-associated oropharyngeal cancer, and HPV vaccine in the United States--do we need a broader vaccine policy?. Vaccine.

[25] Takes R.P., Wierzbicka M., D’Souza G., Jackowska J., Silver C.E., Rodrigo J.P., Dikkers F.G., Olsen K.D., Rinaldo A., Brakenhoff R.H. (2015). HPV vaccination to prevent oropharyngeal carcinoma: What can be learned from anogenital vaccination programs?. Oral Oncol..

[26] Nielsen K.J., Jakobsen K.K., Jensen J.S., Grønhøj C., Von Buchwald C. (2021). The Effect of Prophylactic HPV Vaccines on Oral and Oropharyngeal HPV Infection-A Systematic Review. Viruses.

[27] Jit M., Brisson M., Portnoy A., Hutubessy R. (2014). Cost-effectiveness of female human papillomavirus vaccination in 179 countries: A PRIME modelling study. Lancet Glob. Health.

[28] European Centre for Disease Prevention and Control Vaccine Scheduler. Human Papillomavirus Infection: Recommended Vaccinations. https://vaccine-schedule.ecdc.europa.eu/Scheduler/ByDisease?SelectedDiseaseId=38&SelectedCountryIdByDisease=-1.

[29] Nguyen-Huu N.H., Thilly N., Derrough T., Sdona E., Claudot F., Pulcini C., Agrinier N., HPV Policy working group (2020). Human papillomavirus vaccination coverage, policies, and practical implementation across Europe. Vaccine.

[30] Chief Sanitary Inspectorate Polish Preventive Vaccination Plan 2022. https://www.gov.pl/web/gis/program-szczepien-ochronnych-na-rok-2022.

[31] Augustynowicz A., Bojar I., Borowska M., Bobiński K., Czerw A. (2020). Self-government HPV vaccination programmes in Poland, 2009-2016. Ann. Agric. Environ. Med..

[32] Jeruzal-Świątecka J., Pietruszewska W. (2020). Awareness of Human Papillomavirus and Its Oncogenic Potential in Head and Neck Cancer among Students: Still More Questions than Answers. Int. J. Environ. Res. Public Health.

[33] Jackowska J., Bartochowska A., Karlik M., Wichtowski M., Tokarski M., Wierzbicka M. (2015). The Knowledge of the Role of Papillomavirus-Related Head and Neck Pathologies among General Practitioners, Otolaryngologists and Trainees: A Survey-Based Study. PLoS ONE.

[34] Smolarczyk K., Duszewska A., Drozd S., Majewski S. (2022). Parents’ Knowledge and Attitude towards HPV and HPV Vaccination in Poland. Vaccines.

[35] Smolarczyk K., Pieta W., Majewski S. (2021). Assessment of the State of Knowledge about HPV Infection and HPV Vaccination among Polish Resident Doctors. Int. J. Environ. Res. Public Health.

[36] Milton A.C., Ellis L.A., Davenport T.A., Burns J.M., Hickie I.B. (2017). Comparison of Self-Reported Telephone Interviewing and Web-Based Survey Responses: Findings from the Second Australian Young and Well National Survey. JMIR Ment. Health.

[37] Nationwide Research Panel Ariadna. https://www.panelariadna.pl/.

[38] Pinkas W., Jankowski M., Wierzba W. (2022). Awareness of Head and Neck Cancers: A 2021 Nationwide Cross-Sectional Survey in Poland. J. Clin. Med..

[39] Furman F.M., Zgliczyński W.S., Jankowski M., Baran T., Szumowski Ł., Pinkas J. (2020). The State of Vaccine Confidence in Poland: A 2019 Nationwide Cross-Sectional Survey. Int. J. Environ. Res. Public Health.

[40] Długosz P. (2021). Predictors of Mental Health after the First Wave of the COVID-19 Pandemic in Poland. Brain Sci..

[41] Statistics Poland National Official Register of the Territorial Division of the Country. http://eteryt.stat.gov.pl/eTeryt/english.aspx.

[42] Sulkowska U., Mańczuk M., Przewoźniak K., Koczkodaj P., Przepiórka I., Cedzyńska M., Didkowska J. (2018). Estimating of the number of cancer cases attributed to HPV infections for Poland in 2015. Nowotw. J. Oncol..

[43] Ganczak M., Owsianka B., Korzeń M. (2018). Factors that Predict Parental Willingness to Have Their Children Vaccinated against HPV in a Country with Low HPV Vaccination Coverage. Int. J. Environ. Res. Public Health.

